# Supplementary material for: Molecular Typing, Characterization of Antimicrobial Resistance, Virulence Profiling and Analysis of Whole-Genome Sequence of Clinical Klebsiella pneumoniae Isolates
Source: Antibiotics (Basel). 2020 May 17;9(5):261. doi: 10.3390/antibiotics9050261 (PMC7277670; doi:10.3390/antibiotics9050261)
Supplement: Supplementary file 1 [file antibiotics-09-00261-s001.pdf]

Table S1. Virulence genes revealed in the whole genomes of the isolates.

Sample id\function	ferric yersiniabactin uptake receptor	iron regulatory protein	aerobactin transporter genes	*mrk* cluster (fimbriae)	yersiniabactin	other
P-6				mrkFHIJ		
P-26				mrkABCDHIJ		
P-28				mrkABCDHIJ		
P-29			iucCD, iutA	mrkABCDFHIJ		
P-45				mrkABCDFHIJ		
P-68	fyuA	irp2		mrkABCDFHIJ	ybtAEQSUX	
P-69				mrkABCDFHIJ		
P-75	fyuA	irp2		mrkABCDFIJ	ybtAQSUX	
P-99				mrkABCDFHIJ		
P-108	fyuA	irp1	iucACD, iutA	mrkABCDFIJ	ybtAEQSUX	rmpA
P-115				mrkABCDFHIJ		mceABCDEG HIJ
P-116	fyuA	irp2		mrkABCDFHIJ	ybtAEQSUX	
P-120	fyuA	irp2		mrkABCDFHIJ	ybtAEQSUX	
P-133				mrkABCDFHIJ		
P-134	fyuA	irp2	iucACD, iutA	mrkABCDFHIJ	ybtAEUX	
P-137	fyuA	irp2		mrkABCDFHIJ	ybtAEU	iroBCDN
P-140	fyuA	irp1	iucACD, iutA	mrkABCDFIJ	ybtAEQSUX	rmpA
P-142				mrkABCDFHIJ		
P-152	fyuA	irp1	iucACD, iutA	mrkABCDHIJ	ybtAEQSUX	
P-154	fyuA	irp2		mrkABCDFHIJ	ybtAEQSUX	
P-160	fyuA	irp1, irp2	iucCD, iutA	mrkABDFHIJ	ybtAEQSUX	
P-176	fyuA	irp2		mrkABCDFHIJ	ybtAEQSUX	
P-183				mrkABCDFHIJ		
P-185	fyuA				ybtAEQSUX	
P-187	fyuA	irp2	iucACD, iutA	mrkABCDFHIJ	ybtAEUX	
P-190				mrkADFH		
P-197				mrkAB		
P-200	fyuA	irp1 ,irp2	iucCD, iutA	mrkABCDFIJ	ybtAQSUX	
P-212	fyuA			mrkABH	ybtAEQSU	kfuC
P-223	fyuA		iucCD, iutA	mrkABCDFIJ	ybtAQSUX	
P-226	fyuA	irp1, irp2	iucACD, iutA	mrkABDFHIJ	ybtAEQSUX	
P-234	fyuA	irp2		mrkABCDFHIJ	ybtAEUX	
P-237	fyuA	irp1		mrkABCFHIJ	ybtAEPSUX	kfuABC
P-250				mrkABCDFHIJ		
P-253	fyuA	irp1		mrkABCFHIJ	ybtAEPSUX	kfuABC
P-259	fyuA			mrkABH	ybtAEQSU	kfuC

allS and entB are not shown for the sake of brevity.
